# Physicochemical characterization of Ayurvedic bhasma (*Swarna makshika bhasma*): An approach to standardization

**DOI:** 10.4103/0974-7788.64409

**Published:** 2010

**Authors:** Sudhaldev Mohaptra, C. B. Jha

**Affiliations:** *Department of Rasa Shastra, Faculty of Ayurveda, Institution of Medical Sciences, Banaras Hindu University, India*

**Keywords:** Scanning electron microscope, *Swarna makshika *bhasma**, X-ray diffraction

## Abstract

*Swarna makshika* [SM], a mineral having various therapeutic uses, has been used since long in Ayurveda. The present study was conducted to generate a fingerprint for raw and processed SM using techniques which can be used by pharmacies. Powdered SM was heated in an iron pan by adding lemon juice for 3 days, till liberation of sulfur fumes stopped. *Bhasma* of this *shuddha* SM was obtained by triturating it withit with *shuddha gandhaka* and lemon juice. It was then subjected to heat in 09* *putas*, and for firing in each *puta*, 4 kg cow dung cakes were used. To assure the quality of *bhasma*, *rasa shastra* quality control tests like *nischandratva*, *varitara*, *amla pariksha*, etc., were used. After the *bhasma* complied with these tests, the *bhasma* was analyzed using X-ray Diffraction (XRD) analysis of raw SM and SM *bhasma* revealed that raw SM contains CuFeS_2_, and SM *bhasma* contains Fe_2_O_3_, FeS_2_, CuS and SiO_2_. Scanning Electron Microscope (SEM) studies showed that the grains in SM *bhasma* were uniformly arranged in agglomerates of size 1-2 microns as compared to the raw SM which showed a scattered arrangement of grains of size 6-8 microns. It may be concluded that raw SM is a complex compound which gets converted into a mixture of simple compounds having very small particle size after the particular process of marana. This is the first report of fingerprinting of SM *bhasma* prepared using this particular method.

## INTRODUCTION

*Swarna makshika* [SM] *bhasma* has been used for *pandu* (anemia), *anidra* (insomnia), *apasmara* (convulsions), *mandagni* (*poor digestion*), *kustha* (skin diseases),[1] etc., as well as a potent *rasayana*[2] drug. Generally, SM *bhasma* is prepared in two steps: *shodhana*, by different techniques like fomentation, heating and quenching and roasting, etc.; and *marana*, by *puta* system of heating in different types of *putas*,[3] like *varahaputa, kukkutaputa, gajaputa*, etc.; and *kupipakwa* procedures,[4] etc. During *marana*, bhavana with lemon juice, *kulottha* decoction, *eranda taila, snuhi ksheera*,[5] etc., are given with addition of *shuddha gandhaka* and shuddha hingula,[6] etc., as associated materials.

SM *bhasma* is used as a single constituent formulation or in multi-ingredient formulation. However, there is variation in collection of raw materials and the pharmaceutical procedure followed, which generates the same *bhasma* with different characters. As a result, reproducibility is often not achieved. In many cases, wrong manufacturing and marketing practice leads to the production of inferior quality products, which reduces efficacy or produces safety concerns. In order to minimize variability and to check adulteration, standardization of a *bhasma* is a must.

Ayurvedic texts have described methods for quality control of finished products through different parameters like *nischasndratva*, *varitara*, *nirutha*, *apunarbhava*, etc., to achieve a specific acceptable standard *bhasma*. This study was performed to characterize the *bhasma* using sensitive tools and techniques. These fingerprints generated for the raw material and *bhasma* could be used as standards to for ensuring quality and reproducibility of standards of the medicines.

## MATERIALS AND METHODS

### Swarna makshika processing

This was done using standard procedures and included two steps, namely *Shodhana* and *marana* of *Swarna makshika*.[7] The *Swarna makshika* was procured from the pharmacy (I.M.S., BHU) and lemon juice (sufficient quantity) was obtained from the market

Iron mortar and pestle, charcoal furnace, iron pan, iron ladle, pyrometer, etc.

At first the *Swarna makshika* was powdered in an iron mortar with an iron pestle. A clean and dry iron pan was then heated on a charcoal furnace onto which was poured the powdered *Swarna makshika* and subjected to intense heat with frequent addition of lemon juice till the liberation of sulfur fume stopped and it turned red. The process was completed in 3 days and the final product called *Shodhita Swarna makshika* obtained.

For the *Marana* of *Swarna makshika*[7]*shuddha gandhaka* and lemon juice (q.s.) were procured. Equal amounts of *shuddha Swarna makshika* and *shuddha gandhaka* were triturated with lemon juice till a homogenous paste was formed. After triturating, small pellets of uniform size and thickness were prepared and dried in sunlight. Pellets were kept inside a *sarava* (shallow earthen disc) and another *sarava* was inverted over it. The joint between the two discs was sealed with a rag and mud/*kapad mitti:* a ribbon of fine cloth uniformly smeared with fuller's earth seven times and dried in sunlight.

The properly sealed and dried *samputa* was subjected to *puta* system of heating with 4 kg cow dung cake. The process was repeated using *shuddha gandhaka* in equal proportion to *Swarna makshika* for the first cycle and then in half the proportion for subsequent 8 cycles. *Bhasma* of the desired quality was obtained in 9 *putas*. The *bhasma* obtained from the above process was taken for analysis.

### Analysis using parameters described in Ayurveda texts

The final *bhasma* was analyzed for quality control as described in Ayurvedic texts as follows and found suitable:

1. *Nischandratva:* The *bhasma* was taken in a Petri dish and observed for any luster in daylight through magnifying glass. No luster was observed in the *bhasma*.
2. *Rekhapurnatvam:* A pinch of *bhasma* was taken in between the thumb and index finger and rubbed. It was observed that the *bhasma* entered into the lines of the finger, and was not easily washed out from the cleavage of the lines.
3. *Varitaratavam:* A small amount of the prepared *bhasma* was sprinkled over the still water in a beaker. It was found that the *bhasma* particles floated over the surface of the water.
4. *Nisvadutvam:* The prepared *bhasma* was found to be tasteless when a small amount was kept on the tongue.
5. *Amla pariksha:* A pinch of prepared *bhasma* was mixed with a little amount of *dadhi* (curds) in a clean and dry Petri dish and observed for any color change. No color change of *dadhi* was observed. The same procedure was followed with lemon juice taken in a test tube, and the same result was observed.
6. *Avami:* Ingestion of 5-10 mg of the *bhasma* did not produce any nausea/ vomiting.

### Analysis using modern parameters

The *bhasma* as well as the starting material (*raw swarna makshik*) was also analysed using the following techniques:

1. X-ray diffraction (qualitative)
2. Scanning electron microscopy (qualitative)

### X-ray diffraction study[8]

X-ray diffraction studies were performed in the Regional Research Laboratory (CSIR), Bhubaneswar.

**Figure 1:**
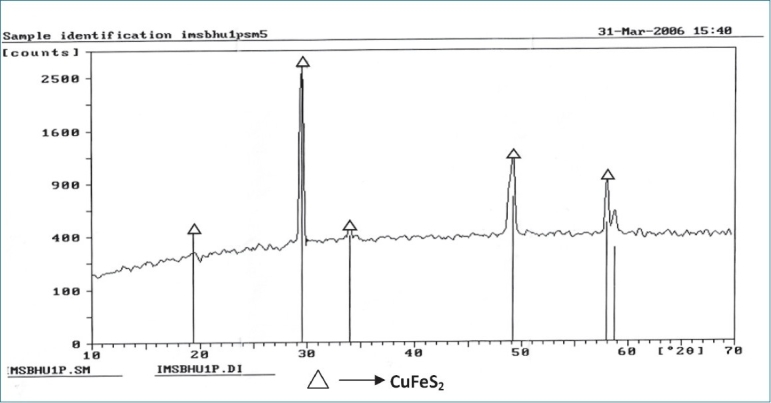
X-ray diffraction of the raw *Swarna makshika*

**Figure 2:**
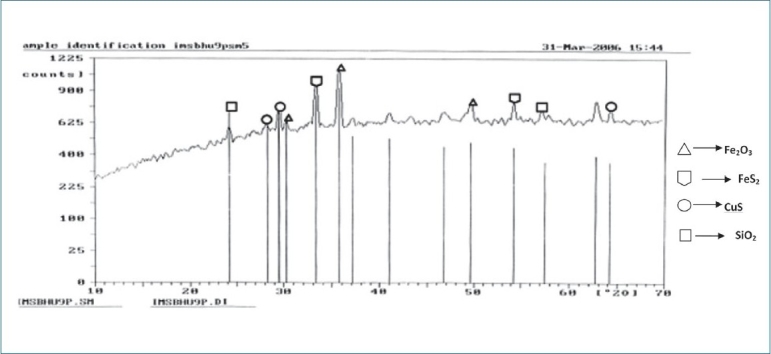
X-ray diffraction of the *Swarna makshika bhasma*

The powdered sample was spread onto a double-side tape with a spatula, which was then placed on an aluminum sample holder. All the peaks were recorded on the chart, and the corresponding 2 theta values were calculated. Results are summarized in Figures 1 and 2 as well as Tables 1 and 2.

**Table 1:** X-ray diffraction of the raw *Swarna makshika*

Compounds	d' value	R. Intensity
CuFeS_2_	3.03	100
	2.64	4
	1.85	21.3
	1.59	12
	1.57	6

**Table 2:** X-ray diffraction of the *bhasma*

Compounds	d' value	R. Intensity
Fe_2_O_3_	2.51	100
	2.95	32.4
FeS_2_	2.69	82. 2
	1.69	24.3
CuS	3.03	50.6
	3.16	26.6
	1.45	12.0
SiO_2_	3.67	31.9
	1.60	8.9

N.B.: 'd' value-'d' space; R. Intensity- Relative intensity

The strongest peak identified in the raw material was CuFeS_2_ while that in the final product was Ferrous oxide of Iron (Fe_2_O_3_). In the raw material, only one phase of Copper iron Sulphide (CuFeS_2_) was identified while in the final product, different phases were identified including Fe_2_O_3_, FeS_2_, Copper sulphide (CuS) and SiO_2_.

### Study using scanning electron microscope[9]

Scanning Electron Microscopy[9] (SEM) was performed in the Dept. of Physics, BHU.

The mounted sample was placed inside the microscope's vacuum column through an airtight door, and then the air was pumped out. After the air was pumped out of the column, a beam of electrons was emitted by an electron gun from the top. This beam travels downward through a series of magnetic lenses designed to focus the electrons to a very fine spot. Near the bottom, a set of scanning coils made the focused beam to move back and forth across the mounted sample, row by row.

**Figure 3:**
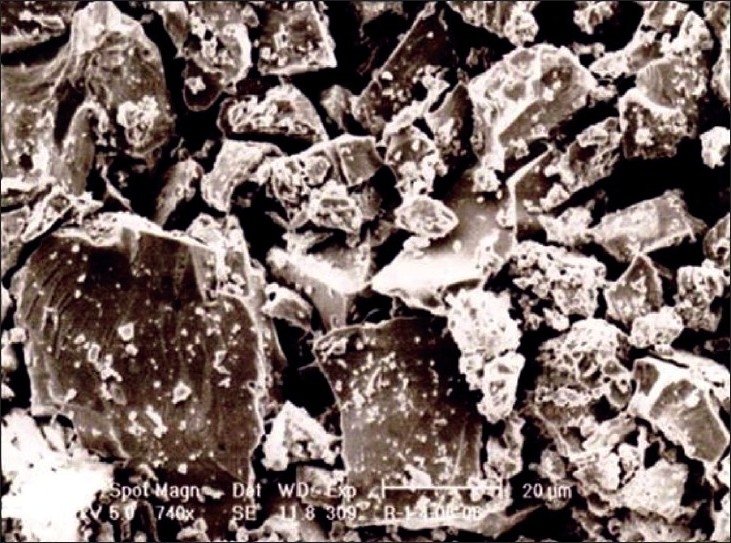
Scanning electron microscopy feature of raw *Swarna makshika*

**Figure 4:**
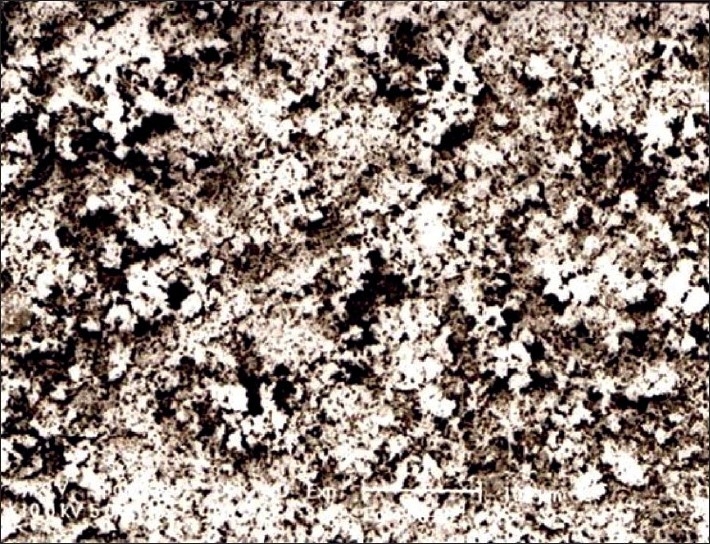
Scanning electron microscopy feature of *Swarna makshika bhasma*

As the electron beam hits each spot on the sample, secondary electrons are backscattered from its surface. A detector counts these electrons and sends the signals to an amplifier. The final image was built up from the number of electrons emitted from each spot on the sample [Figures 3 and 4].

The particles in the raw material and in the sample of intermediary process were not uniformly arranged while in the final product the particles uniformly arranged. In the final product, more agglomerates of grain were observed, whereas in the raw material the grains were found scattered. The size of the particles was less in the final product.

The particle size of the raw material was between 6 and 8 µ while that for the *bhasma* particles was 1-2 µ.

## DISCUSSION

It is noteworthy that there are very specific pharmaceutical procedures and techniques described in the *rasa shastra* literature which convert the toxic metals/ minerals into a suitable dosage form. The *bhasmas* prepared are well tolerated both for short-term and long-term use; moreover, it is claimed that their prolonged administration is required to achieve the rejuvenation effect.[10] According to the need of time, characterization of *bhasma* using scientific techniques is necessary to determine the effect of the process and to judge its safety and efficacy. SM *bhasma* was prepared and studied with this objective. X-ray diffraction study of the raw material showed a sharp peak, indicating its crystalline nature; whereas the final product did not give sharp peaks, indicating the loss of crystalline nature. This is suggested by the test described in Ayurveda namely, as loss of luster *(nischandrika)* in the final product. Thus the sharp crystalline structure of the raw material reflects light rays whereas loss of crystalline nature in the final product prevents it from doing so.Hence the "loss of luster" *(nischandrika)*described in Ayurveda as a quality to be looked for in the final product.

The study also revealed peaks of CuFeS_2_ in the raw material and Fe_2_O_3_, Iron sulphide (FeS_2_), CuS and silicon oxide (SiO_2_) in the final product. The formation of some different compounds in the final product may be due to oxidation and reduction reaction of Cu, Fe with sulfur in the presence of oxygen. It is likely that the lack of change of colour in an acidic medium (*amla pariksha*) in the final product is due to the absence of free metallic groups as free copper reacts with lemon juice to give a blue color. Sulfides and oxides of iron and copper present in *makshika bhasma* do not show any unwanted effect in experimental study,[11] and the *bhasma* has been used over a long period of time in clinical practice and no toxic effect has been recorded so far. Presence of SiO_2_ may be due to the use of earthen casseroles, which may have a reaction with oxygen. Scanning electron microscopy study showed that the particle size reduced from 6-8 µ to 1-2 µ after *bhasma* process. It is this significant reduction of size and that allows the phenomenon of *rekhapurna* and *varitara* to develop. Reduction in particle size facilitates absorption and assimilation of the *bhasma* in the system. Again the clusters of particles are regular and uniform in the final *bhasma* in comparison to the raw material. The particle size recorded can be characterized as the desired specification of the final *bhasma*.

## CONCLUSION

Thus, *Swarna makshika* which contains iron (Fe), Copper (Cu) and sulfur The manufacturing process plays a specific role to convert the CuFeS_2_ in the raw material mixture of Fe_2_O_3_, FeS_2_, CuS and SiO_2_, in the final product. These could be important chemical markers for SM *bhasma* prepared using this particular method. As a result of different stages of processing techniques like *shodhana* (which involves roasting, with addition of herbal juices and continuous stirring) and *marana* [which involves bhavana (wet trituration) and *puta* system of heating], the particle size reduces significantly, which may facilitate absorption and assimilation of the drug into the body system. The particle size in the final *bhasma* was 1-2 µ, which could be specified as the criterion for the final product conforming to all the traditional parameters under *bhasma pariksha* (examination of properly prepared *bhasma*). This can be one of the important factors for standardization of *bhasmas*.Thus, modern techniques can assist in proper characterization of *Ayurvedic* dosage forms and standardization of *Ayurvedic* medicines.

## GLOSSARY OF IMPORTANT TERMS USED IN THIS ARTICLE

### Tests of bhasma

*Varitara*- The *bhasma* that floats on water is termed as *varitara*.

*Apunarbhava- Bhasma* when mixed with *mitrapanchaka* and heated at high temperature should not undergo any change in its physical properties. The *bhasma* should not regain its original state.

*Niruttha- Bhasma* is heated at high temperature in a *koshthi* along with measured quantity of silver. At the end of the process, the quantity of silver should not increase.

*Niswadu- Bhasma* should be tasteless. If *bhasma* has any taste, it is considered as semi-finished and should be subjected to *puta* again.

*Nischandra*- The sparkling particles (*chandrika*) in a *bhasma* indicate a semi-finished product.

*Avami*- The *bhasma* should not produce nausea on administration.

*Puta*- In continuation with the etymological meaning, *puta* is the measure of the amount of heat required to convert or transform any metal or mineral. This amount is substance specific and measured in terms of number or weight of fuel.

*Sharava*- Earthen Petri dish having specific measurements.

*Bhavana*- Trituration of the drug with liquid medium, e.g., hingula with juice of fresh zinzibar officinalis.
